# Rice Bran Phenolic Extracts Modulate Insulin Secretion and Gene Expression Associated with β-Cell Function

**DOI:** 10.3390/nu12061889

**Published:** 2020-06-24

**Authors:** Nancy Saji, Nidhish Francis, Lachlan J. Schwarz, Christopher L. Blanchard, Abishek B. Santhakumar

**Affiliations:** 1 Australian Research Council (ARC) Industrial Transformation Training Centre (ITTC) for Functional Grains, Graham Centre for Agricultural Innovation, Charles Sturt University, Wagga Wagga, NSW 2650, Australia; nsaji@csu.edu.au (N.S.); nfrancis@csu.edu.au (N.F.); lschwarz@csu.edu.au (L.J.S.); CBlanchard@csu.edu.au (C.L.B.); 2 School of Biomedical Sciences, Charles Sturt University, Locked Bag 588, Wagga Wagga, NSW 2678, Australia; 3 School of Animal and Veterinary Sciences, Charles Sturt University, Locked Bag 588, Wagga Wagga, NSW 2678, Australia; 4 School of Agricultural and Wine Sciences, Charles Sturt University, Locked Bag 588, Wagga Wagga, NSW 2678, Australia

**Keywords:** rice bran, phenolic extracts, β-cell function, gene expression, insulin secretion

## Abstract

Oxidative stress is known to modulate insulin secretion and initiate gene alterations resulting in impairment of β-cell function and type 2 diabetes mellitus (T2DM). Rice bran (RB) phenolic extracts contain bioactive properties that may target metabolic pathways associated with the pathogenesis of T2DM. This study aimed to examine the effect of stabilized RB phenolic extracts on the expression of genes associated with β-cell function such as glucose transporter 2 (*Glut2*), pancreatic and duodenal homeobox 1 (*Pdx1*), sirtuin 1 (*Sirt1*), mitochondrial transcription factor A (*Tfam*), and insulin 1 (*Ins1*) in addition to evaluating its impact on glucose-stimulated insulin secretion. It was observed that treatment with different concentrations of RB phenolic extracts (25-250 µg/mL) significantly increased the expression of *Glut2*, *Pdx1*, *Sirt1, Tfam,* and *Ins1* genes and glucose-stimulated insulin secretion under both normal and high glucose conditions. RB phenolic extracts favourably modulated the expression of genes involved in β-cell dysfunction and insulin secretion via several mechanisms such as synergistic action of polyphenols targeting signalling molecules, decreasing free radical damage by its antioxidant activity, and stimulation of effectors or survival factors of insulin secretion.

## 1. Introduction

Glucose homeostasis is regulated by a sequence of events within the pancreatic β-cells, which result in the secretion of insulin [1]. Typically, in the postprandial state, increased levels of glucose in plasma can initiate pancreatic β-cells to secrete insulin, consequently suppressing hepatic glucose output and increasing peripheral tissue glucose uptake [2]. However, impairment of glucose-stimulated insulin secretion as a result of oxidative stress and inflammation can result in β-cell dysfunction and insulin resistance, subsequently leading to the pathogenesis of type 2 diabetes mellitus (T2DM) [3]. There are several essential genes involved in insulin secretion pathways that are specifically expressed in pancreatic β-cells. They are known to be involved in the processes leading to insulin release from the initial glucose entry into the β-cells followed by mitochondrial adenosine triphosphate (ATP) generation and potassium and calcium membrane depolarization leading to exocytosis events [4]. They include, glucose transporter 2 (*Glut2*) [5], pancreatic and duodenal homeobox 1 (*Pdx1*) [6], sirtuin 1 (*Sirt1*) [7], mitochondrial transcription factor A (*Tfam*) [8], and insulin 1 (*Ins1*) [9].

The *Glut2* gene is located in the pancreatic plasma membrane and functions as a glucose transporter as part of the glucose-sensing mechanism for the stimulation of insulin secretion [2]. The *Pdx1* gene plays an important role in mitochondrial embryonic development and β-cell differentiation and is known to regulate the expression of a variety of different pancreatic endocrine genes, including *Glut2* [10]. The *Sirt1* gene is known to serve as a key energy redox sensor involved in generating ATP that helps promote glucose-stimulated insulin secretion in pancreatic β-cells and potentially contribute to β-cell adaptation in response to insulin resistance [1]. In the liver, skeletal muscles, and white adipose tissues, the *Sirt1* gene has key functions that include regulation of glucose production, improvement in insulin sensitivity via fatty acid oxidation, and control of the production of adipokines [7]. The *Tfam* gene plays an essential role in the maintenance of mitochondrial DNA (mtDNA) and replication [11]. Altered mitochondrial function is known to result in a defective oxidative metabolism, which seems to be involved in visceral fat gain and the development of insulin resistance [12]. Moreover, the *Tfam* gene is also involved in insulin exocytosis events by maintaining appropriate ADP/ATP ratio [4]. The *Ins1* gene and its transcription factors are regulated by the circulating levels of glucose [9]. It encodes the production of insulin that plays a vital role in the regulation of carbohydrate and lipid metabolism [13]. Therefore, a disruption in the function of these genes (*Glut2*, *Pdx1*, *Sirt1*, *Tfam,* and *Ins1*) in the pancreatic β-cells is known to impair insulin secretion and result in the development of T2DM.

Recent studies have shown the potential of plant-derived phenolic compounds in ameliorating β-cell dysfunction via their antioxidant and free radical scavenging properties [14,15,16]. Exposure of polyphenols to β-cells has also been responsible for the modulation of several signalling proteins, including transcription factors, protein kinase, and ion channels [17].

Rice bran (RB), a by-product of the rice milling process, is usually discarded or used as animal feed [18]. However, the bran layer is composed of several bioactive phytochemicals, including polyphenols and phenolic acids [19]. Although RB phenolic extracts are believed to target metabolic pathways associated with T2DM, the mechanisms behind its effect on gene expression under normal and diabetic conditions have not been investigated. This study aimed to determine the effect of RB phenolic extracts on the expression of genes (*Glut2*, *Pdx1*, *Sirt1*, *Tfam,* and *Ins1*) associated with insulin secretion pathways and on glucose-stimulated insulin secretion under normal and high glucose conditions.

## 2. Materials and Methods

### 2.1. Chemicals and Reagents

All chemicals and reagents used in this study were purchased from Promega Corporation (Madison, WI, USA), Bio-Rad (Hercules, CA, USA), or Sigma-Aldrich (St Louis, MO, USA).

### 2.2. Rice Bran Phenolic Extract Preparation

Commercially stabilized RB (drum-dried), from an Australian grown Reiziq rice variety, was obtained from SunRice Australia, courtesy of their milling plant in Leeton, NSW, Australia and subsequently stored at 4 °C until further analysis. Phenolic compounds were extracted from stabilized RB using an acetone/water/acetic acid (70:29.5:0.5, v/v) mixture, the characterization of which has been described elsewhere [18]. It is known to contain several bioactive compounds including ferulic acid, p-coumaric acid, caffeic acid, vanillic acid, syringic acid, sinapic acid, feruloyl glycoside, shikimic acid, ethyl vanillate, tricin, and their isomers. The extract was reconstituted in 50% dimethyl sulfoxide (DMSO) and stored at −20 °C before starting cell culture studies.

### 2.3. Cell Culture Conditions

INS-1E cells were maintained in RPMI 1640 media containing 11.1 mM glucose and supplemented with 2 mM L-Glutamine, 1 mM sodium pyruvate, 10 mM HEPES, 0.05 mM β-mercaptoethanol, 10% Fetal Bovine Serum, and 1% 10,000 U/mL Penicillin–10 mg/mL Streptomycin from Sigma-Aldrich (St Louis, MO, USA) at 37 °C in 5% CO_2_ and used before reaching passage 45.

### 2.4. Cytotoxicity Assay

The cytotoxicity of RB phenolic extracts was examined using a resazurin red cytotoxicity assay wherein INS-1E cells were seeded into 96-well plates at a density of 50,000 cells per well and incubated for 24 h in the RPMI 1640 complete media. The cell count for experimental seeding was achieved with a Muse^®^ Cell Analyzer from Luminex Corporation (Austin, TX, USA). INS-1E cells were then treated with 200 µL of freshly prepared RB phenolic extracts at various concentrations (25, 50, 100, 250, 500, 750, and 1000 µg/mL) for 6 h. Hydrogen peroxide (5 mM) was used as a positive control and 0.25% DMSO served as a negative control. Subsequently, all the treatment wells were emptied before adding 200 µL of resazurin red solution (14 mg/L) to each well and incubated for an additional 4 h at 37 °C in 5% CO_2_. The absorbance was measured on a microplate reader (FLUOstar Omega microplate reader, BMG Labtech, Offenburg, Germany) at 570 and 600 nm against a resazurin red blank. The percentage of cell viability was calculated as described by Saji, Francis [20]. Each treatment was measured in octuplicate.

### 2.5. Expression of Genes Associated with β-Cell Function

#### 2.5.1. Experimental Design

Two experimental conditions simultaneously tested were normal glucose treatment (11.1 mM) to represent a normal β-cell function and high glucose treatment (25 mM) to represent β-cell dysfunction under glucotoxic stress [4,15]. INS-1E cells were seeded at a density of 500,000 cells per well into 6-well plates and incubated for 24 h. To induce glucotoxicity, INS-1E cells were further subjected to 48 h incubation in RPMI 1640 complete media containing 25 mM glucose. Cells under both normal and high glucose conditions were treated for 6 h with RB phenolic extracts (25, 50, 100, and 250 µg/mL) and 0.125% DMSO served as the negative control. Each treatment was measured in quintuplicate.

#### 2.5.2. Gene Expression Analysis

Total ribonucleic acid (RNA) extraction was conducted using the SV Total RNA Isolation System according to the manufacturer’s instructions (Promega, Madison, WI, USA). RNA quality was determined using a NanoDrop™ 2000 c Spectrophotometer from Thermo Fisher Scientific (Waltham, MA, USA). Then, cDNA synthesis was conducted using a GoScript™ Reverse Transcriptase, according to the manufacturer’s instructions (Promega, Madison, WI, USA).

Primers used for quantitative real-time polymerase chain reaction (qPCR) examinations are listed in Table 1. All of the qPCR primers were adapted from [21], designed using Primer3 software, and synthesized by Sigma-Aldrich (St Louis, MO, USA). The amplification efficiency was determined to be between 90–110% for all the primers before starting qPCR.

Gene expression was conducted in the CFX96 Touch™ Real-Time PCR Detection System (Bio-Rad) using SsoAdvanced™ Universal SYBR^®^ Green Supermix (Bio-Rad) detection according to the manufacturer’s instructions. The cycling conditions comprised 95 °C for 3 min, 95 °C for 10 s, and 60 °C for 30 s repeated for 39 cycles. The melt curve was generated at 65 °C for 5 s and 95 °C for 50 s. The endpoint or cycle threshold (Ct) values were obtained for all genes tested. The mean normalized expression of genes was determined using the Q-gene software application, as described by Muller, Janovjak [22]. *TfIIβ* served as the reference gene.

### 2.6. Glucose-Stimulated Insulin Secretion

The preparation of supernatant for the glucose-stimulated insulin secretion assay was adapted from a previous study conducted by Bhattacharya, Oksbjerg [15] with slight modifications. Briefly, INS-1E cells were seeded into a 24-well plate at a density of 1 × 10^5^ cells/well and incubated for 24 h or until 70–80% confluency was reached. The cells were treated with DMSO control and RB extracts at different concentrations (25–250 µg/mL) and incubated for 6 h. Cells were then starved with a Krebs-Ringer bicarbonate buffer (125 mM NaCl, 5.9 mM KCl, 1.28 mM CaCl_2_, 1.2 mM MgCl_2_, 25 mM HEPES, and 0.1% BSA at pH 7.4) containing 5 mM glucose for 1 h. Glucose-stimulated insulin secretion was then induced by treating cells with a Krebs-Ringer bicarbonate buffer containing either 11.1 mM or 25 mM glucose for 1 h. The supernatant containing secreted insulin was collected and stored at −20 °C until further analysis. Insulin secretion was measured using a Rat *Ins1*/Insulin ELISA Kit purchased from Sigma-Aldrich (St Louis, MO, USA) according to the manufacturer’s instructions. Each treatment was measured in sextuplicate.

### 2.7. Statistical Analysis

Statistical analysis was performed by one-way analysis of variance (ANOVA), followed by post-hoc Tukey’s multiple comparisons test using GraphPad Prism 7 software (GraphPad Software Inc, San Diego, CA, USA) at a level of *p* < 0.05. The results are reported as mean ± standard deviation (SD).

## 3. Results

### 3.1. Cytotoxicity of RB Phenolic Extracts on INS-1E Cells

**Figure 1:**
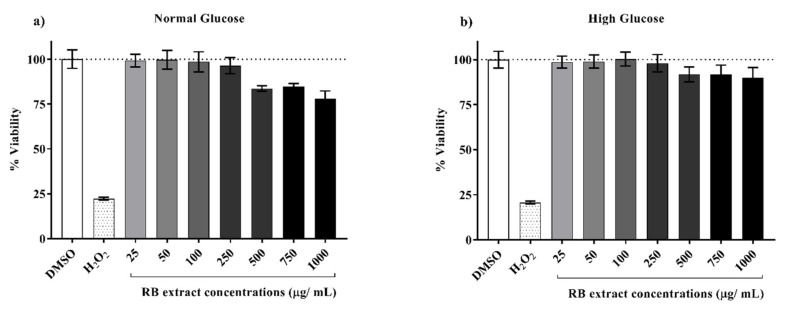
INS-1E cell viability 6 h post-exposure to various concentrations of RB phenolic extracts. (**a**) Normal glucose conditions, (**b**) High glucose conditions (*n* = 8). Data are presented as Mean ± SD. Dimethyl sulfoxide, DMSO; Hydrogen Peroxide, H_2_O_2_; Insulin-secreting rat insulinoma cell, INS-1E; Rice bran, RB.

The cell viability of INS-1E cells 6 h post-exposure (Figure 1) to various concentrations of RB phenolic extracts did not display any cytotoxic effect on the INS-1E cells at the lower concentrations tested (25–250 µg/mL). However, higher concentrations (500–1000 µg/mL) displayed a reduction in cell viability. Optimal, non-toxic concentrations of RB extract were determined to be between 25–250 µg/mL under both normal and high glucose conditions.

### 3.2. Effect of RB Phenolic Extracts on Expression of Genes Associated with β-Cell Function

#### 3.2.1. Expression of the *Glut2* Gene

**Figure 2:**
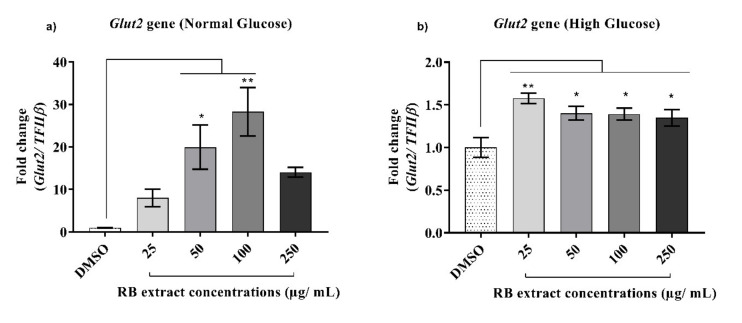
Effect of RB phenolic extracts on the expression of the *Glut2* gene under normal and high glucose conditions in INS-1E cells. (**a**) Normal glucose conditions, (**b**) High glucose conditions (*n* = 5). The level of significance is indicated by the asterisks, whereby * *p* < 0.05, ** *p* < 0.01. Data are presented as Mean ± SD. Dimethyl sulfoxide, DMSO; Insulin-secreting rat insulinoma cell, INS-1E; Glucose transporter 2, *Glut2*; Rice bran, RB.

Under normal glucose conditions, a significant increase (*p* < 0.01) in the expression of the *Glut2* gene was observed after treatment with 50 and 100 µg/mL of RB phenolic extracts when compared to that of the control. Under high glucose conditions, a significant increase (*p* < 0.0001) in the expression of the *Glut2* gene was also observed after treatment with 25–250 µg/mL of RB phenolic extracts when compared to that of the control (Figure 2).

#### 3.2.2. Expression of the *Pdx1* Gene

**Figure 3:**
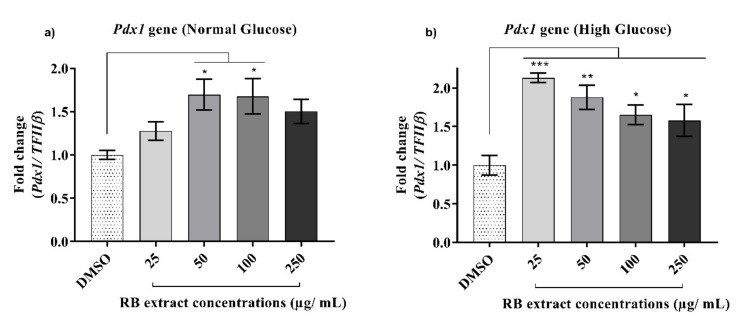
Effect of RB phenolic extracts on the expression of the *Pdx1* gene under normal and high glucose conditions in INS-1E cells. (**a**) Normal glucose conditions, (**b**) High glucose conditions (*n* = 5). The level of significance is indicated by the asterisks, whereby * *p* < 0.05, ** *p* < 0.01, *** *p* < 0.001. Data are presented as Mean ± SD. Dimethyl sulfoxide, DMSO; Insulin promoter factor 1, *Pdx1*; Insulin-secreting rat insulinoma cell, INS-1E; Rice bran, RB.

A significant increase (*p* < 0.05) in the expression of the *Pdx1* gene was observed after treatment with 50 and 100 µg/mL of RB phenolic extracts under normal glucose conditions when compared to that of the control. A significant increase (*p* < 0.001) in the expression of the *Pdx1* gene was also observed after treatment with 25–250 µg/mL of RB phenolic extracts under high glucose conditions when compared to that of the control (Figure 3).

#### 3.2.3. Expression of the *Sirt1* Gene

**Figure 4:**
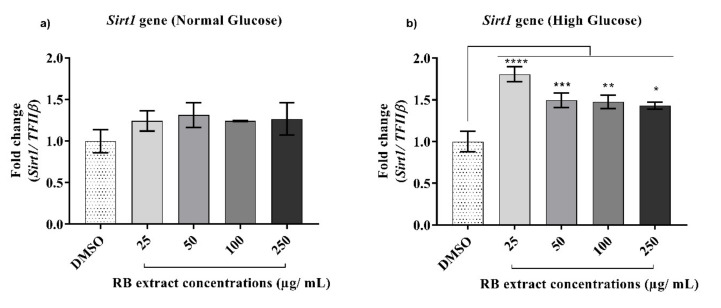
Effect of RB phenolic extracts on the expression of the *Sirt1* gene under normal and high glucose conditions in INS-1E cells. (**a**) Normal glucose conditions, (**b**) High glucose conditions (*n* = 5). The level of significance is indicated by the asterisks, whereby * *p* < 0.05, ** *p* < 0.01, *** *p* < 0.001, **** *p* < 0.0001. Data are presented as Mean ± SD. Dimethyl sulfoxide, DMSO; Insulin-secreting rat insulinoma cell, INS-1E; Rice bran, RB; Sirtuin 1, *Sirt1*.

There was no significant increase in the expression of the *Sirt1* gene observed under normal glucose conditions when compared to that of the control. However, under high glucose conditions, a significant increase (*p* < 0.0001) in the expression of the *Sirt1* gene was observed after treatment with 25–250 µg/mL of RB phenolic extracts when compared to that of the control (Figure 4).

#### 3.2.4. Expression of the *Tfam* Gene

**Figure 5:**
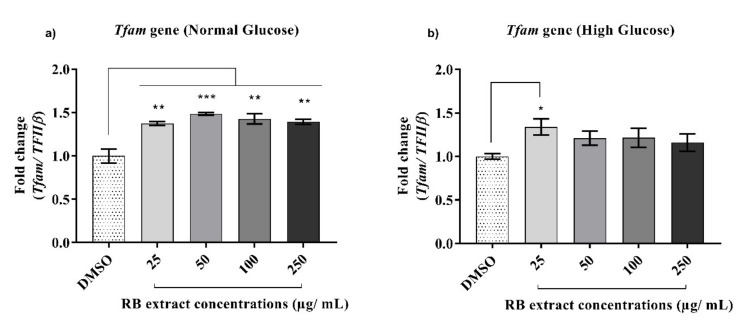
Effect of RB phenolic extracts on the expression of the *Tfam* gene under normal and high glucose conditions in INS-1E cells. (**a**) Normal glucose conditions, (**b**) High glucose conditions (*n* = 5). The level of significance is indicated by the asterisks, whereby * *p* < 0.05, ** *p* < 0.01, *** *p* < 0.001. Data are presented as Mean ± SD. Dimethyl sulfoxide, DMSO; Insulin-secreting rat insulinoma cell, INS-1E; Mitochondrial transcription factor A, *Tfam*; Rice bran, RB.

Under normal glucose conditions, a significant increase (*p* < 0.001) in the expression of the *Tfam* gene was observed after treatment with 25–250 µg/mL of RB phenolic extracts when compared to that of the control. However, under high glucose conditions, a significant increase (*p* < 0.05) in the expression of the *Tfam* gene was only observed after treatment with 25 µg/mL of RB phenolic extracts when compared to that of the control (Figure 5).

#### 3.2.5. Expression of the *Ins1* Gene

**Figure 6:**
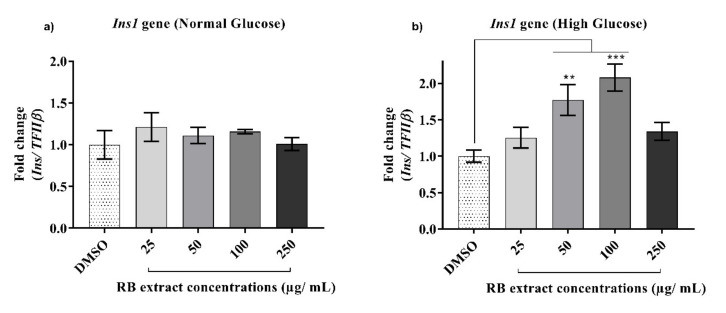
Effect of RB phenolic extracts on the expression of the *Ins1* gene under normal and high glucose conditions in INS-1E cells. (**a**) Normal glucose conditions, (**b**) High glucose conditions *(n* = 5). The level of significance is indicated by the asterisks, whereby ** *p* < 0.01, *** *p* < 0.001. Data are presented as Mean ± SD. Dimethyl sulfoxide, DMSO; Insulin 1, *Ins1*; Insulin-secreting rat insulinoma cell, INS-1E; Rice bran, RB.

RB extract did not alter the expression of the *Ins1* gene under normal glucose treatment. However, under high glucose conditions, a significant increase in the expression of the *Ins1* gene was observed after treatment with 50 µg/mL (*p* < 0.01) and 100 µg/mL (*p* < 0.001) of RB phenolic extracts when compared to that of the control (Figure 6).

### 3.3. Glucose-Stimulated Insulin Secretion

**Figure 7:**
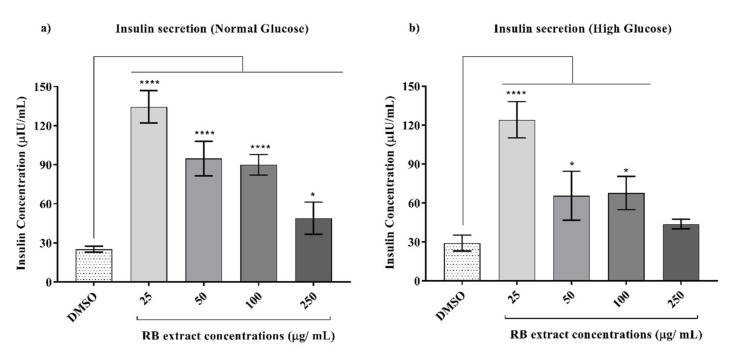
Effect of RB phenolic extracts on glucose-stimulated insulin secretion under normal and high glucose conditions in INS-1E cells. (**a**) Normal glucose conditions, (**b**) High glucose conditions (*n* = 6). The level of significance is indicated by the asterisks, whereby * *p* < 0.05, **** *p* < 0.0001. Data are presented as Mean ± SD. Dimethyl sulfoxide, DMSO; Insulin-secreting rat insulinoma cell, INS-1E; Rice bran, RB.

Glucose-stimulated insulin secretion was observed to significantly increase after treatment with 25 (*p* < 0.0001), 50 (*p* < 0.0001), 100 (*p* < 0.0001), and 250 (*p* < 0.05) µg/mL of RB phenolic extracts under normal glucose conditions when compared to that of the control. Similarly, under high glucose conditions, a significant increase in glucose-stimulated insulin secretion was also observed after treatment with 25 (*p* < 0.0001), 50 (*p* < 0.05), and 100 (*p* < 0.05) µg/mL of RB phenolic extracts when compared to that of the control (Figure 7).

## 4. Discussion

Prolonged exposure of pancreatic β-cells to a high glucose environment is known to result in oxidative stress, consequently leading to the downregulation of pancreatic genes, in turn causing impaired β-cell function and insulin secretion [16]. Plant-derived phenolic compounds via their antioxidant, free radical scavenging and metal chelating properties have been observed to target metabolic pathways associated with the pathogenesis of T2DM [14]. The present study demonstrated that RB phenolic extracts effectively alter β-cell function in insulin-secreting cells by modulating the expression of genes and insulin secretion. It was observed that RB phenolic extracts upregulated the expression of key genes associated with β-cell function, including *Glut2*, *Pdx1*, *Sirt1*, *Tfam,* and *Ins1* both under normal and high glucose-induced stress conditions (Figure 2, Figure 3, Figure 4, Figure 5 and Figure 6).

The *Glut2* gene primarily acts as a glucose transporter and the decreased expression of the *Glut2* gene is directly proportional to the loss of glucose-stimulated insulin secretion [5]. In this study, a significant increase in the expression of the *Glut2* gene was observed under normal conditions compared to that in high glucose conditions. This may have been caused by the increase in glucotoxic stress created by the high glucose environment, resulting in a reduced ability to maintain normal functioning as a glucose transporter. Nevertheless, a significant up-regulation of the *Glut2* gene was observed under both conditions compared to those of the respective controls after treatment with varying concentrations of RB extract (Figure 2). Similarly, studies in which phenolic compounds derived from *M. pumilum var. alata* extracts and purified phenolic compounds such as resveratrol were tested improved β-cell function, and insulin signalling was observed as a result of increased expression of the *Glut2* gene in the pancreas [21,23]. This is most likely due to the polyphenols targeting the exchange of calcium ions resulting in the exocytosis of insulin-containing granules, thereby favourably modulating β-cell function [5,24].

*Pdx1* gene expression is essential for the homeostatic regulation of the glucose-sensing system in β-cells [6]. It is also essential for survival and differentiation of β-cells as it primarily acts by upregulating the transcription of several β-cell-specific genes, including the *Ins* and *Glut2* genes [25]. Results obtained from this study show that under both normal and high glucose conditions, a significant upregulation of the *Pdx1* gene was evident after treatment with RB phenolic extracts (Figure 3). Upregulation of the *Pdx1* gene has been observed elsewhere, in which administration of *Teucrium polium* extract, known to contain phenolic compounds with strong antioxidant and anti-inflammatory effects, was found to reverse the symptoms of streptozotocin-induced diabetes in rats [26]. Another study, wherein the effect of gallic acid against glucolipotoxicity and insulin secretion was examined, showed that pre-treatment with different concentrations of gallic acid was found to increase insulin secretion and resulted in the upregulation of the *Pdx1* gene in RINm5F β-cells [27]. Reduction in insulin secretion has been attributed to the c-Jun N-terminal kinase (JNK) pathway activation under oxidative stress conditions. JNK activation as a result of oxidative stress results in forkhead box protein O1 (FOXO1) phosphorylation, and the nuclear localization of the FOXO1protein leads to a reduction in the expression of the *Pdx1* gene [28]. As an adequate expression of the pancreatic *Pdx1* gene is essential to maintain the proper function of insulin-producing β-cells, inhibition of the JNK pathway is crucial. As phenolic compounds are recognized to modulate the regulation of the JNK pathway [26], it is likely that the observed upregulation of the *Pdx1* gene by RB-derived phenolic extracts resulted from an inhibition of the JNK pathway.

The *Sirt1* gene is known to be a major contributor to the metabolic regulation of a cell via lipid metabolism and insulin secretion [7]. In the current study, under high glucose conditions, a significant increase in the expression of the *Sirt1* gene was observed after treatment with RB phenolic extracts (Figure 4). Sun, Zhang [29] demonstrated that resveratrol improved insulin sensitivity by repressing the protein tyrosine phosphatase (PTP) constitute and PTP_1_B transcription at the chromatin level (on the *Sirt1* gene) under normal versus insulin-resistant conditions. Hence, it is believed that upregulation of the *Sirt1* gene as a result of treatment with RB phenolics can potentially target PTP_1_B ranscription consequently improving insulin sensitivity.

Any disruption to the *Tfam* gene in the pancreatic β-cell is known to result in impaired insulin secretion, reduced β-cell mass, and, consequently, the development of T2DM [8]. The current study shows a significant increase in the expression of the *Tfam* gene under normal and high glucose conditions post-treatment with RB phenolic extracts (Figure 5). In an in vivo study where rats were gavaged with pterostilbene, *Tfam* gene expression was significantly increased in addition to improvements to glycaemic control and insulin resistance [30]. Furthermore, the treatment of INS-1E cells with resveratrol also displayed marked potentiation of glucose-stimulated insulin secretion as a result of the up-regulation of *Tfam* [21]. From the above studies, it is believed that RB phenolics have the potential to enhance the efficiency of mitochondrial function via interaction with transcription factors such as *Tfam*.

Appropriate regulation of the *Ins1* gene is essential for central insulin signalling as it is an anorectic gene that encodes for the production of the insulin hormone that plays a vital role in the regulation of carbohydrate and lipid metabolism [31]. Chronic exposure to high glucose conditions can reduce the expression of the *Ins1* gene in β-cells and is often accompanied by the decreased binding activity of the β-cell-specific transcription factor, *Pdx1* [32]. In the current study, although there was no significant increase in *Ins1* gene expression after RB extract treatment under normal glucose conditions, the expression of the *Ins1* gene was significantly upregulated under high glucose conditions (Figure 6). Similarly, an in vivo study by the author of [33] also demonstrated blueberry-leaf extract rich in chlorogenic acid and flavonol glycosides attenuates glucose homeostasis and improves pancreatic β-cell function by increasing the expression of several genes including *Ins1.* Polyphenols present in common spices, such as cinnamon, cloves, turmeric, and bay leaves, due to their doubly-linked procyanidin type-A polymers, have also shown an insulin-like activity in vitro [34]. The mechanism of cinnamon’s insulin-like activity may be in part due to increases in the amounts of insulin receptor β and *Glut4* expression [34]. Some of the polyphenols present in cinnamon include caffeic, ferulic, *p*-coumaric, protocatechuic, and vanillic acids [35], a similar phenolic profile observed in the RB samples used in this study [18]. Therefore, it is likely that the effects observed in this study may be due to the insulin-like activity displayed by the polyphenols present in RB individually or via synergistic bioactivity.

Hormones such as insulin and amylin are co-secreted by β-cells in a fixed molecular ratio that provides circulating energy in the form of glucose and stored energy in the form of visceral adipose tissue [36]. However, conditions such as obesity, T2DM, and pancreatic cancer result in an increase in the amount of amylin relative to the insulin, which can disturb the regulation of energy homeostasis [36]. It was observed that under normal and high glucose-induced conditions, RB phenolic extracts significantly increased glucose-stimulated insulin secretion (Figure 7). Bhattacharya, Oksbjerg [15] also observed a similar trend where caffeic acid, naringenin, and quercetin significantly increased glucose-stimulated insulin secretion under hyperglycaemic and glucotoxic conditions in INS-1E cells. Similarly, several other phenolic compounds such as ferulic acid [37] and *p*-coumaric acid [38] have also been shown to increase insulin secretion both in vitro and in vivo, respectively. In this study, it was observed that RB phenolic compounds increase the expression of both the *Ins1* gene and the secretion of insulin in INS-1E cells under high glucose conditions. Since the *Ins1* gene is known to encode for the production of insulin hormone, this may indicate that there may be a correlation between insulin secretion and the expression of the *Ins1* gene.

Furthermore, it was observed that lower doses of the RB extract used in this study favourably modulated β-cell function associated gene expression and insulin secretion when compared to the higher doses in vitro. This phenomenon may be explained through the effect of hormesis, a biphasic dose-response to an environmental agent, wherein glucose-stimulated insulin secretion was observed to have a stimulatory or beneficial effect at low doses and an inhibitory or toxic effect at high doses of RB extract [39]. Dietary polyphenols are known to have strong cytoprotective effects, however, the hormetic role of dietary antioxidants in free radical-related diseases have demonstrated that under uncontrolled nutritional supplementation, gene induction effects and the interaction with detoxification responses can result in a negative response by generating more reactive and harmful intermediates [40].

As a result of hindrance by cereal matrices, most of the bound phenolic compounds present in cereal grains are usually not readily accessible by digestive enzymes, leading to low bioavailability [41]. Studies have demonstrated that this could be improved by increasing their accessibility through suitable processing techniques, for example, thermal treatments [18,41]. The RB sample examined in this study was previously studied with respect to several thermal treatments. Of the treatments studied, drum drying resulted in the optimal antioxidant activity and was therefore selected for the current investigation [18]. The drum-dried RB samples resulted in a total free phenolic content of 362.17 ± 34.16 gallic acid equivalent (GAE)/100 g of RB with antioxidant activity of 975.33 ± 20.24 Fe^2+^/100 g of RB and a total bound phenolic content of 160.65 ± 5.52 GAE/100 g of RB with antioxidant activity of 551.91 ± 8.82 Fe^2+/^100 g of RB. This was much higher compared to that of a non-treated sample that had a total free phenolic content of 238.26 ± 30.34 GAE/100 g of RB with antioxidant activity of 621.76 ± 26.76 Fe^2+/^100 g of RB and a total bound phenolic content of 222.94 ± 3.74 GAE/100 g of RB with antioxidant activity of 712.37 ± 14.57 Fe^2+^/100 g of RB [18].

## 5. Conclusions

This study has demonstrated that RB phenolic compounds, under both normal and glucotoxic conditions, significantly increase the expression of genes associated with β-cell function, in addition to increasing glucose-stimulated insulin secretion. RB phenolic compounds could play an important role in modulating the expression of genes involved in β-cell dysfunction and insulin secretion via several mechanisms, including (1) Synergistic action of polyphenols and phenolic acids by targeting signalling molecules, including transcription factors, consequently modulating mitochondrial potential; (2) Reducing free radical damage related to β-cell dysfunction via their antioxidant activity; and (3) Stimulation of effectors or survival factors of insulin secretion. RB phenolic extracts present as a promising preventive/therapeutic target in the treatment of glucotoxicity induced β-cell dysfunction. More in vivo studies are warranted to confirm the bioactivity of RB phenolic compounds.

## Figures and Tables

**Table 1:** The nucleotide sequences of the PCR primers used to assay gene expression by qPCR.

Gene	Forward Primer	Reverse Primer
***Glut2***	TCAGCCAGCCTGTGTATGCA	TCCACAAGCAGCACAGAGACA
***Pdx1***	CCGCGTTCATCTCCCTTT C	CTCCTGCCCACTGGCTTT T
***Sirt1***	CAGTGTCATGGTTCCTTTGC	CACCGAGGAACTACCTGA T
***Tfam***	GGGAAGAGCAAATGGCTGAA	TCACACTGCGACGGATGA GA
***Ins1***	TGCTCACCCGCGACCTT	GTTCATATGCACCACTGGACTGAA
***TfIIβ***	GTTCTGCTCCAACCTTTGCCT	TGTGTAGCTGCCATCTGCACT T

## Abbreviations

c-Jun N-terminal kinase (JNK), Dimethyl sulfoxide (DMSO), Forkhead box protein O1 (FOXO1), Gallic acid equivalent (GAE), Glucose transporter 2 (*Glut2*), Insulin 1 (*Ins1*), Insulin-secreting rat insulinoma cell (INS-1E), Mitochondrial DNA (mtDNA), Mitochondrial transcription factor A (*Tfam*), One-way analysis of variance (ANOVA), Pancreatic and Duodenal Homeobox 1 (*Pdx1*), Protein tyrosine phosphatase (PTP), Quantitative real-time polymerase chain reaction (qPCR), Ribonucleic acid (RNA), Rice bran (RB), Sirtuin 1 (*Sirt1*), Standard deviation (SD), Type 2 diabetes mellitus (T2DM).
